# Tumour‐derived substrate‐adherent cells promote neuroblastoma survival through secreted trophic factors

**DOI:** 10.1002/1878-0261.12969

**Published:** 2021-05-06

**Authors:** Jing Li, Yubing Wang, Lisha Li, Penelope M.‐Y. Or, Chi Wai Wong, Tian Liu, Wayne L.H. Ho, Andrew M. Chan

**Affiliations:** ^1^ School of Biomedical Sciences The Chinese University of Hong Kong Hong Kong SAR China; ^2^ Present address: School of Bioscience and Technology Weifang Medical University China

**Keywords:** ALK, neuroblastoma, STAT3, substrate‐adherent cells, tumour microenvironment

## Abstract

Neuroblastoma (NB) is the most common extracranial solid tumour in children. NB is highly heterogeneous and is comprised of a mixture of neuroblastic cancer cells and stromal cells. We previously reported that N‐type cells (neuroblastic cells) and S‐type cells (substrate‐adherent cells) in the SK‐N‐SH cell line shared almost identical genetic backgrounds. Sublines of N‐ and S‐type cells were isolated from an early passage (P35) of SK‐N‐SH. Sequencing analysis revealed that all sublines harboured the anaplastic lymphoma kinase (ALK) F1174L mutation, indicating that they were tumour derived. Surprisingly, over 74% resembled S‐type cells. In coculture experiments, S‐type cells protected N‐type cells from apoptosis induced by the oncogenic ALK inhibitor TAE684. Western blotting analyses showed that ALK, protein kinase A (AKT) and STAT3 signalling were stimulated in the cocultures. Furthermore, the conditioned medium from S‐type cells activated these downstream signalling molecules in the N‐type cells. The activation of STAT3 in the N‐type cells was ALK‐independent, while AKT was regulated by the ALK activation status. To identify the responsible soluble factors, we used a combination of transcriptomic and proteomic analysis and found that plasminogen activator inhibitor 1, secreted protein acidic and cysteine rich, periostin and galectin‐1 were potential mediators of STAT3 signalling. The addition of recombinant proteins to the tumour cells treated with the ALK inhibitor partially enhanced cell viability. Overall, the tumour‐derived S‐type cells prevented apoptosis in the N‐type cells *via* ALK‐independent STAT3 activation triggered by secreted factors. The inhibition of these factors in combination with ALK inhibition could provide a new direction for targeted therapies to treat high‐risk NB.

## Graphical abstract

Neuroblastoma S‐type cells promote survival of N‐type tumor cells through cell‐to‐cell contact, which is associated with upregulation of p‐STAT3 in N‐type cells. Independently, S‐type cells secrete trophic factors that promote the survival of N‐type cells. Trophic factors could act either by stimulating p‐STAT3, or by upregulating p‐AKT and p‐ERK1/2 through an p‐ALK‐dependent pathway. The inhibition of these factors in combination with ALK inhibition could provide a new strategy for treatment of high‐risk neuroblastoma.

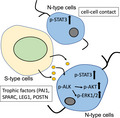

**Abbreviations**
AKT: protein kinase A
ALK: anaplastic lymphoma kinase
CFSE: carboxyfluorescein succinimidyl ester
CM: conditioned medium
DEGs: differential expressed genes
GRN: granulins
*GRP78*
: glucose‐regulated protein 78
IFC: integrated fluidic circuits
*LEG1*
: galectin‐1
NB: neuroblastoma
*PAI1*
: plasminogen activator inhibitor 1
*POSTN*
: periostin
rhIL‐6: recombinant human interleukin‐6
SPARC: secreted protein acidic and cysteine rich
TAE684: NPV‐TAE684

## 1 Introduction

Neuroblastoma (NB) is the most common extracranial solid tumour in children, and the median age at diagnosis is 18 months [1]. It is widely accepted that NB arises from the sympathoadrenal lineage of the neural crest during development [2]. The most common primary sites of NB are the adrenal glands, and the sympathetic ganglia distributed along the paraspinal areas from the neck to the pelvis [3]. Approximately 60% of NB cases present with metastatic disease, mainly of the bone marrow, bone, lymph nodes, liver and skin [4]. The International NB Risk Group stratifies patients into four groups: very low risk, low risk, intermediate risk and high risk [5]. In terms of therapy, the very low and low‐risk groups are commonly treated with observation and surgical resection [6]. In the intermediate‐risk group, chemotherapy followed by surgery is common. For the high‐risk group, a protocol comprising of induction, myeloablative consolidation and maintenance is often adopted. New antitumour drugs, such as targeted therapeutics, are considered. Examples of targets that are under investigation are MYCN, ornithine decarboxylase, Aurora A kinase, TRKA/B, histone deacetylases and ALK [7].

Anaplastic lymphoma kinase belongs to the superfamily of receptor tyrosine kinases and was first discovered as a mutated oncogene in familial NBs [8]. Oncogenic *ALK* mutations account for 8–12% of all somatic NB cases. The binding of a ligand to a receptor results in the dimerization of ALK receptors and stimulates downstream signalling by the trans‐autophosphorylation of Tyr1278, Tyr1282 and Tyr1283 [9]. Several ligands have been identified for ALK, including pleiotrophin [10], midkine [11], heparin [12], FAM 150A and FAM 150B [13, 14]. Activating ALK mutations induce the constitutive phosphorylation of ALK downstream signalling pathways. The two major mutations that have been identified in NBs are R1275Q and F1174L, which enhance the activation of ALK by fourfold and eightfold, respectively, when compared to the ligand‐dependent activation of wild‐type ALK [15]. The ALK downstream pathways include the PLCγ, JAK/STAT3, PI3K/protein kinase A (AKT) and MAPK signalling pathways. Several small‐molecule inhibitors have been developed that target oncogenic ALK, including NPV‐TAE684 (TAE684), crizotinib, ceritinib, alectinib, brigatinib and lorlatinib [16, 17, 18, 19].

In the tumour microenvironment of NB, stromal cells such as endothelial cells, pericytes, and cancer‐associated fibroblasts all contributed to the progression of NB [20]. The presence of Schwannian stroma in NB is age‐ and stage‐related and may be related to the differentiation or maturation process in neuroblastic tumours [21]. Based on their stromal abundance, neuroblastic tumours are classified as NBs; ganglioneuroblastomas, intermixed; ganglioneuromas; or ganglioneuroblastomas, nodular. All stroma‐rich or stroma‐dominant tumours with maturation potential are associated with favourable clinical outcomes, except for ganglioneuroblastomas, nodular. In NBs, however, Schwannian stromal‐poor tumours are frequently associated with unfavourable outcomes.

The bone marrow is a frequent site for metastatic growth in high‐risk NB, and mesenchymal stromal cells (MSCs) play a prominent role in the survival and chemoresistance of NB in the bone marrow microenvironment [22]. MSCs have been implicated in both protumorigenic and tumour‐suppressing functions. Following multimodal treatment, residual disease in bone marrow is responsible for tumour relapse and MSCs may provide a favourable niche for tumour cell dormancy and subsequent recurrence [23]. In this study, we investigated sublines from early passage SK‐N‐SH cell line, which was originally derived from bone marrow metastasis of a high‐risk NB patient. These sublines are positive for several stromal cell markers, and they possess prosurvival activities towards anti‐ALK drug. By transcriptomic and proteomic analysis, we identified several secreted factors that are candidates in mediating these processes.

## 2 Methods

### 2.1 Cell lines and cell culture

The human NB cell line, SK‐N‐SH (HTB‐11), was purchased from the American Type Culture Collection (ATCC, Manassas, VA, USA). N‐type cell (NC3) and S‐type cell (S1, S2, S3, S4, S5, S6) clones were generated from the SK‐N‐SH line. LA1‐55n and LA1‐5s cell lines were gifts from R. A. Ross (Fordham University, NY, USA). For clonal derivation, the SK‐N‐SH cell suspension was diluted to 10 cells·mL^−1^, and the cells were seeded into a 96‐well plate. The wells were monitored every 2 days, and wells with a single cell clone were selected. All cells were cultured in Dulbecco's Modified Eagle's medium (DMEM) with 10% heat‐inactivated FBS and 100 unit·mL^−1^ penicillin–streptomycin (Thermo Fisher Scientific, Waltham, MA, USA). All cell lines were propagated at 37 °C in a 5% CO_2_ atmosphere. For conditioned medium (CM) preparation, S‐type cells at 80% confluence were cultured with DMEM/10%FBS for 2 days. Control CM was prepared by adding DMEM/10%FBS to a blank plate and incubated for the same duration in a CO_2_ incubator.

### 2.2 Reagents and antibodies

MTS and Stattic were purchased from Sigma‐Aldrich (St Louis, MO, USA), and PMS was obtained from Promega (Madison, WI, USA). TAE684 was purchased from Selleckchem (Houston, TX, USA). Cell‐labelling carboxyfluorescein succinimidyl ester (CFSE) and recombinant human secreted protein acidic and cysteine rich (SPARC) protein were from Abcam (Cambridge, UK). The human recombinant protein IL‐6 (R&D System, Minneapolis, MN, USA), and galectin‐1 (LEG1), periostin (POSTN) and plasminogen activator inhibitor (PAI‐1) proteins (Sino Biological, Beijing, China) were purchased from the indicated commercial sources. The IL‐6 neutralizing antibody (501109) was purchased from Bio Legend (San Diego, CA, USA). The antibodies for p‐ALK Tyr1604 (#3341), ALK (#3633), p‐JAK2 Tyr 221 (#3774), JAK2 (#3230), p‐STAT3 Tyr705 (#4113), STAT3 (#4904), p‐AKT Ser473 (#4051), AKT (#9272), Erk1/2 (#9102), p‐Erk1/2 Thr202/Tyr204 (#9106) and GAPDH (#5174), anti‐rabbit horseradish peroxidase (HRP)‐conjugated secondary (#7074) and anti‐mouse HRP‐conjugated secondary (#7076) were obtained from Cell Signaling Technology (Danvers, MA, USA). The antibodies for NF68 (Ab72995) and S100α6 (Ab134149) were purchased from Abcam. The HRP‐conjugated anti‐actin (sc‐1615) and HRP‐conjugated anti‐goat IgG (sc‐2352) antibodies were from Santa Cruz Biotechnology (Dallas, Texas, USA).

### 2.3 Single cell analysis

Cell capture and targeted preamplification were conducted using Fluidigm C1 machine and integrated fluidic circuits (IFC; Fluidigm, San Francisco, CA, USA). Briefly, priming solutions were infused into IFC using a C1 machine, and then, SK‐N‐SH cell suspension was loaded. After visually confirming the presence of a single cell in each well, lysis, reverse transcription and 18 cycles of preamplification were conducted in the IFC using a C1™ Single Cell Reagent Kit for Preamp (Fluidigm). Amplified products were harvested from IFC and transferred into 25 µL of DNA suspension buffer (Teknova, Hollister, CA, USA). Sample and assay mixtures were loaded onto a 48.48 Dynamic Array IFC for standard amplification in a Biomark machine as per manufacturer's instructions (Fluidigm) using an Ambion Single Cell‐to‐C_T_ kit (Thermo Fisher Scientific). Data were analysed with singular analysis toolset Software v3.6.2. (Fluidigm).

### 2.4 RNA sequencing

Total RNA was extracted using an RNA extraction kit (Omega Bio‐Tek, Norcross, GA, USA), and then, quantitative RNA sequencing was performed by BGI (Beijing, China) using the Ion Torrent platform. The raw reads were first filtered using soapnuke (v1.5.2) software (BGI) with the parameters: ‐l 15 ‐q 0.5 ‐n 0.1 to remove the low‐quality reads (> 20% of the base qualities were < 10), reads with adaptors and reads with unknown bases (N bases > 5%). Clean reads were then assembled into Unigenes, followed with Unigene functional annotation, SSR detection and calculated the Unigene expression levels and SNPs of each sample. The clean reads were mapped using HISAT (Hierarchical Indexing for Spliced Alignment of Transcripts) to the reference genome hg38 downloaded from the University of California, Santa Cruz (http://genome.uscs.edu/). The gene expression levels were quantified using the software package Sailfish. The reads per kilobase of exon model per million reads method was used to calculate the gene expression level. The differential expressed genes (DEGs) between the S‐ and N‐type cells were determined by the analysis method based on a Poisson distribution. The genes with an expression log_2_ value larger than 2 and a *P*‐value of < 0.05 were filtered and considered as significant DEGs.

### 2.5 Mass spectrometry

Conditioned media collected were spun at 3900 ***g*** at 4 °C for 10 min. A 3 kDa cut‐off concentration column (Merck Millipore, Burlington, MA, USA) was then used to concentrate the CM at 3900 ***g*** at 4 °C for 2 h. Around 50 mL of CM was concentrated down to 300 µL in 50 mm ammonium bicarbonate buffer. Protein samples (10 µg) were loaded on an SDS/PAGE and stained with Coomassie Blue (Bio‐Rad, Hercules, CA, USA). The stained bands were excised and followed by in‐gel digestion using an In‐Gel Tryptic Digestion Kit (Thermo Fisher Scientific). After digestion, the samples were dried in a vacuum concentrator. The peptide pellets were analysed by mass spectrometry on a Dionex Ultimate 3000 Nano‐LC system (Thermo Fisher Scientific), Bruker Proteineer fc II and Bruker UltrafleXtreme MALDI‐TOF‐TOF mass spectrometer (Bruker, Billerica, MA, USA) at the Proteomics Core of The Chinese University of Hong Kong.

### 2.6 Statistical analysis

The experimental data are presented as the mean ± SD. All experiments were repeated at least three times unless indicated otherwise. graphpad prism 6 (San Diego, CA, USA) was used for statistical analysis. The variance among the groups was analysed using Student's *t*‐test, one‐way ANOVA and two‐way ANOVA, as noted. *P*‐values are denoted as follows: * *P* < 0.05, **P < 0.01, ***P < 0.001, ****P < 0.0001.

## 3 Results

### 3.1 Establishment of N‐ and S‐type sublines from SK‐N‐SH

**Fig. 1:**
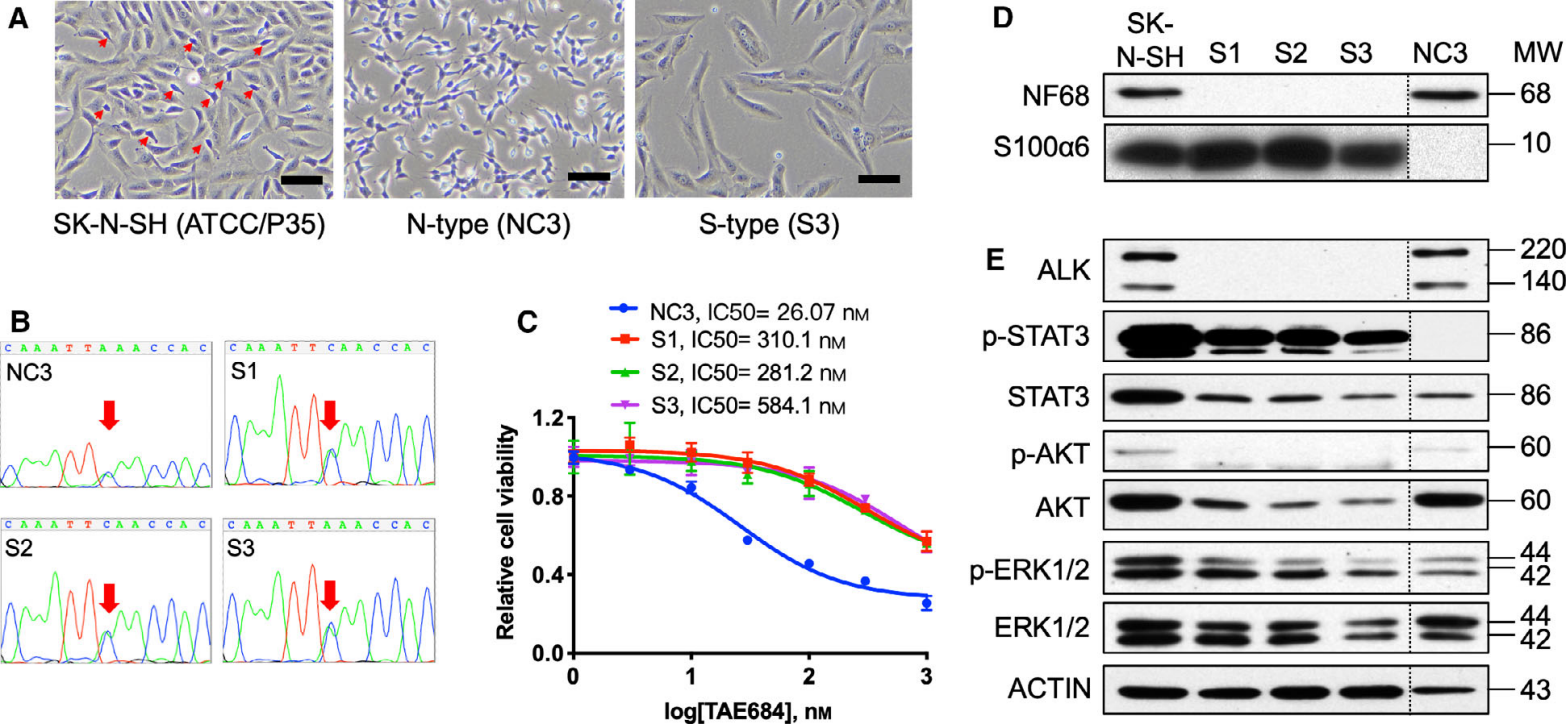
Characterization of NB subtypes. (A) Cell morphology of SK‐N‐SH (N‐type cells, red arrows), NC3 and S3 cells are shown. Bars, 100 μm. (B) ALK genomic regions from NC3, S1, S2 and S3 cells were sequenced to reveal the C to A substitution at codon 1174 (red arrows). (C) Around 2 × 10^4^ NC3, and 3 × 10^3^ S1, S2 or S3 cells were plated per well of 96‐well plates in triplicates and treated with an increasing concentration of TAE684 for 48 h. Cell viability was measured by the MTS assay. Estimated IC50 values are indicated. Error bars, SD. (D) Expression of neuronal marker NF68 and stromal marker S100α6 in indicated cell types. (E) The status of ALK and its downstream signalling pathways, including STAT3, AKT and ERK was analysed in the indicated types by western blotting analysis using the respective antibodies. Actin was used as a loading control. [Colour figure can be viewed at wileyonlinelibrary.com]

The SK‐N‐SH cell line was established from the bone marrow metastases of a patient with a high‐risk NB that possessed the *ALK* F1174L mutation. Morphologically, SK‐N‐SH is composed of neuroblastic N‐type cells and substrate‐adherent S‐type cells [24]. Previously, our laboratory established several S‐type sublines that were insensitive to the ALK inhibitor, TAE684 [25]. To further elucidate the molecular mechanism underlying the crosstalk between N‐ and S‐type cells, we re‐established both N‐ and S‐type sublines using an earliest available passage of SK‐N‐SH at 35 (P35) from the American Type Culture Collection (ATCC). The P35 SK‐N‐SH culture was predominantly populated with ~ 74% S‐type cells based on morphological criteria (Fig. 1A, Fig. S1A,B). Next, one N‐type subline, NC3, and six S‐type sublines, S1–S6, were established (Fig. 1A). S‐type sublines have variable proliferative potential with S3 subline displayed greater proliferation and S5 ceased to proliferate after multiple passages. Cell lines with genomic DNA available (S1, S2, S3, S4 and S6) were subjected to Sanger sequencing analysis, and the ALK F1174L mutation was detected in all these sublines (Fig. 1B and Fig. S1C). These data reaffirm that the S‐type cells from the SK‐N‐SH are tumour derived [25].

To test the relative sensitivity of these cell lines to ALK inhibition, individual sublines were treated with TAE684 for 48 h. The N‐type cells were hypersensitive to TAE684 treatment, with an IC_50_ of 26 nm (Fig. 1C). Meanwhile, all three S‐type cells were less sensitive to TAE684, with IC_50_ values 10‐ to 22‐fold higher than those of N‐type cells. Western blotting analysis revealed N‐type cells expressed the neural marker, Neurofilament 68 (NF68), while all S‐type sublines expressed the S100 alpha 6 (S100α6/calcyclin) as previously described (Fig. 1D) [26]. The presence of the two ALK isoforms in only N‐type cells could explain their hypersensitivities to TAE684 (Fig. 1E). N‐type cells display moderate levels of phosphorylated AKT (p‐AKT) and ERK1/2 (p‐ERK1/2) but very low levels of p‐STAT3. In contrast, S‐type cells have robust p‐STAT3 expression (Fig. 1E).

**Fig. 2:**
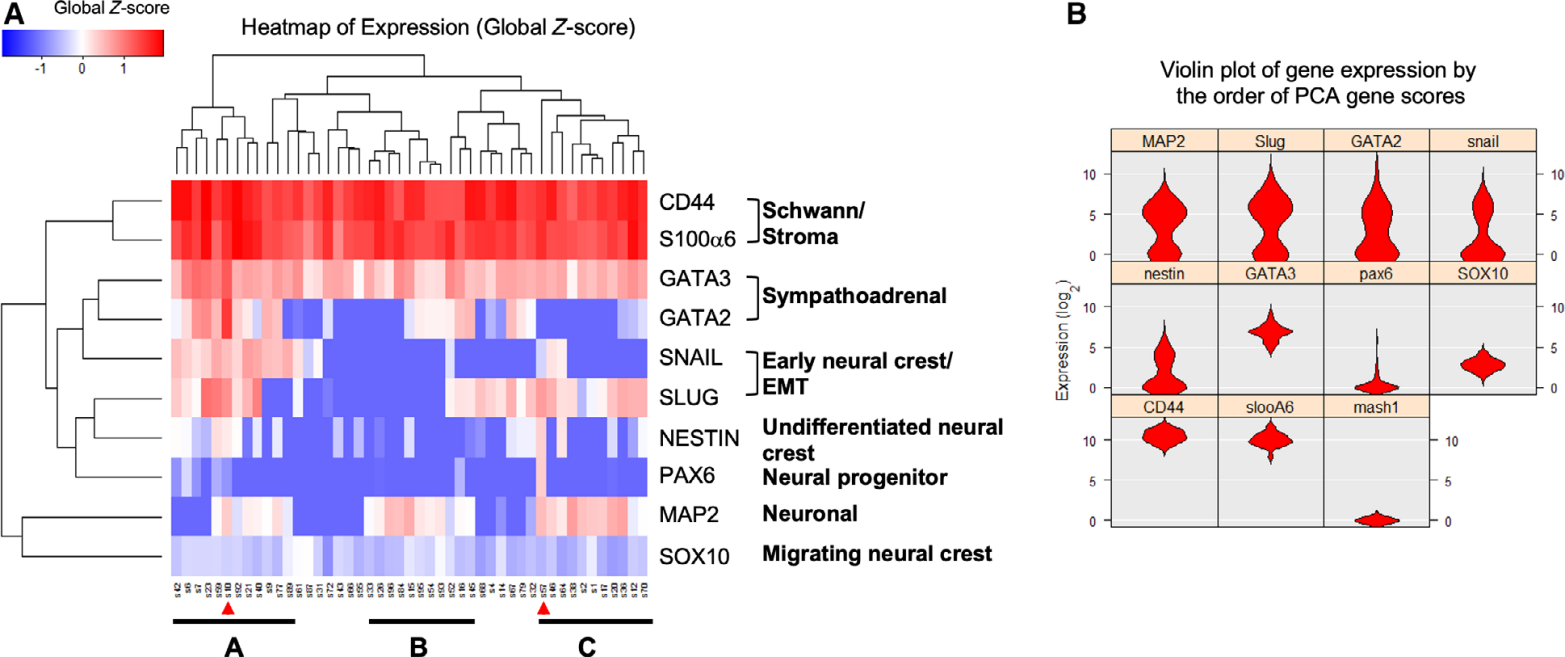
Cellular heterogeneity of SK‐N‐SH cell line. (A) Gene expression heatmap of single cells based on global *Z*‐score for the indicated lineage‐specific markers. (B) Violin plot of gene expression by the order of PCA gene scores. Figures were generated using singular software (Fluidigm). [Colour figure can be viewed at wileyonlinelibrary.com]

To examine the heterogeneity of the P35 SK‐N‐SH culture, 47 single cells were captured and subjected to gene expression analysis using genes related to neural crest development. A gene expression heatmap revealed that most cells have readily detectable levels of CD44 and S100α6, which are substrate‐adherent cell markers (Fig. 2A). Single cells were clustered into three subtypes: Group A was enriched with genes related to early neural crest; Group B had preferential expression of neuronal markers; and Group C had high expression of both early neural crest and neuronal markers. Furthermore, two cells displayed high expression of stem cell markers, GATA2 and Pax6, suggesting that they were cancer stem‐like cells (red arrowheads; Fig. 2A). A violin plot of gene expression further showed that MAP2, SLUG, GATA2 and SNAIL were widely distributed, indicating the high heterogeneity of SK‐N‐SH cells (Fig. 2B).

### 3.2 S‐type cells protected N‐type cells from apoptotic and necrotic cell death by cocultivation

**Fig. 3:**
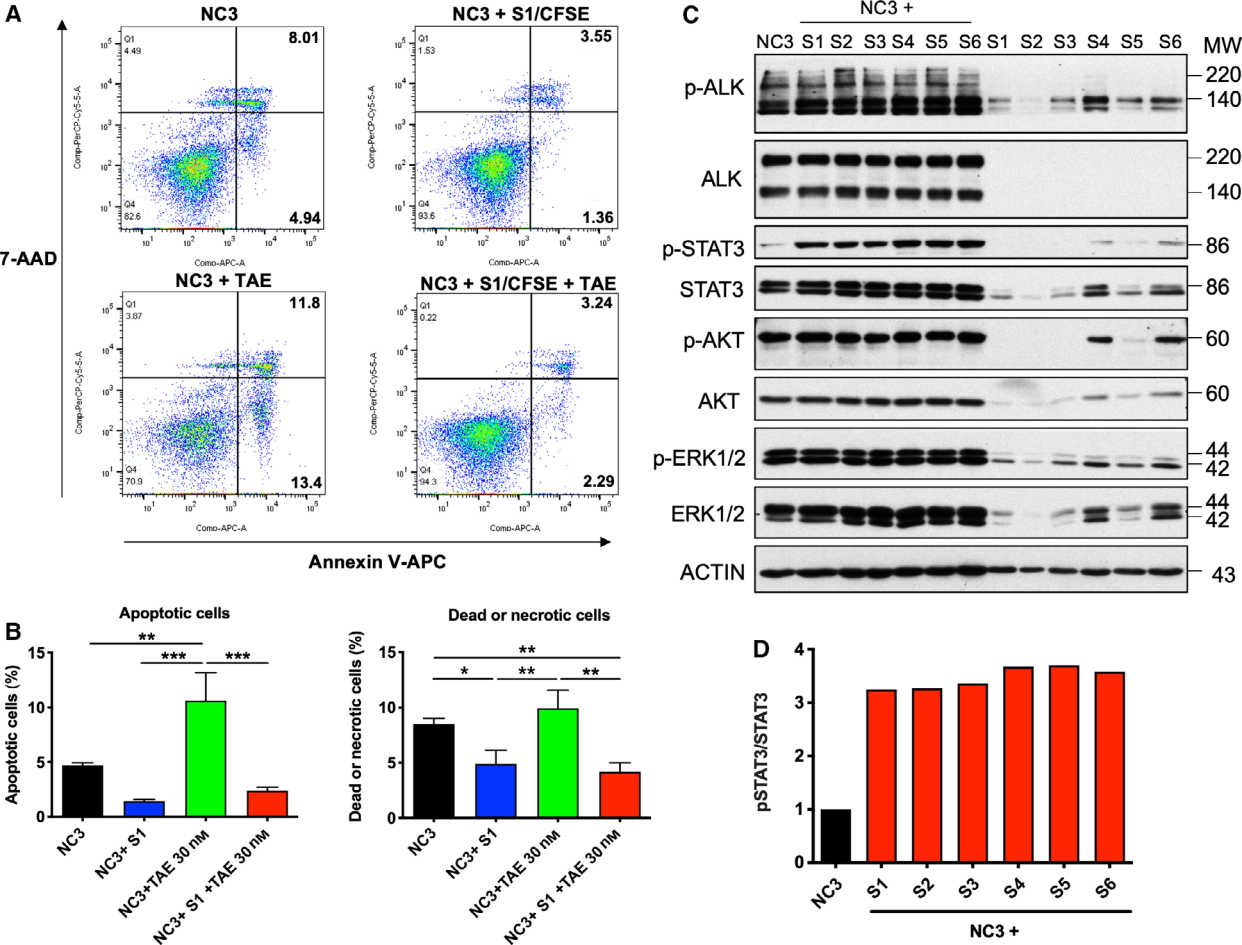
S‐type cells protect N‐type cells from apoptosis. (A) 3 × 10^5^ NC3 cells were comixed with or without 1 × 10^5^ CFSE‐labelled S1 cells per well of 60‐mm plates and were treated with 30 nm TAE684 for 48 h. Cells were stained with APC‐annexin V and 7‐AAD. CFSE‐negative populations (NC3 cells) were analysed for fractions of AnV^+^ apoptotic cells (lower right quadrants) and AnV^+^ 7‐AAD^+^ dead or necrosis cells (upper right quadrants). (B) Results from 3 independent experiments were quantified using flowjo (BD, San Jose, CA, USA). Statistics, one‐way ANOVA. Error bars, SD. *P < 0.05, **P < 0.01, ***P < 0.001. (C) Approximately 8 × 10^5^ NC3 cells were comixed with 3 × 10^4^ S1–S6 cells in a ratio of 80 : 3 per well of six‐well plates. After 48 h, cells were lysed and western blotting analysis was conducted with antibodies indicated. (D) The relative phosphorylation of the p‐STAT3 was quantified using imagelab software (BIO‐RAD, Hercules, CA, USA). [Colour figure can be viewed at wileyonlinelibrary.com]

As S‐type cells were insensitive to TAE684, their ability to protect N‐type cells from anti‐ALK treatment was tested by a coculturing method. The S‐type cells were labelled with a cell tracer dye, CFSE. The N‐type cells were comixed with or without S‐type cells and treated with 30 nm TAE684. The NC3 cells had an intrinsic apoptotic rate of 4.94%, which was reduced to 1.36% when cocultured with the S1 cells (Fig. 3A,B). Similarly, the comixing with S1 cells decreased the dead or necrotic population (upper right quadrant in Fig. 3A) from 8.01% to 3.55%. The N‐type cells treated with 30 nm TAE684 had a higher apoptotic population (13.4%) and a higher dead or necrotic population (11.8%). Coculturing with the S1 cells significantly reduced the rates of the apoptotic and dead or necrotic populations to 2.29% and 3.24%, respectively (Fig. 3A,B). Similar results were obtained with S2 and S3 cells (Fig. S2). Additional S‐type cells from other NB cell lines were tested. A threefold decrease in dead or necrotic cell death was observed when S‐type cells were cocultured with LA1‐5S (Fig. S3).

The signalling alterations in the N‐type cells after comixing with the S‐type cells were analysed (Fig. 3C,D). Around 8 × 10^5^ N‐type cells were comixed with 3 × 10^4^ S‐type cells for 48 h. The low number of S‐type cells that were used favoured the detection of the signalling events in N‐type cells. Coculture upregulated the phosphorylation of STAT3 by threefold. In contrast, the levels of p‐ALK, p‐AKT and p‐ERK1/2 were not elevated (Fig. 3C,D). These results suggest that the anti‐apoptotic function probably relied on the stimulation of STAT3 signalling.

### 3.3 S‐type cell‐conditioned medium promoted N‐type cell viability and proliferation

**Fig. 4:**
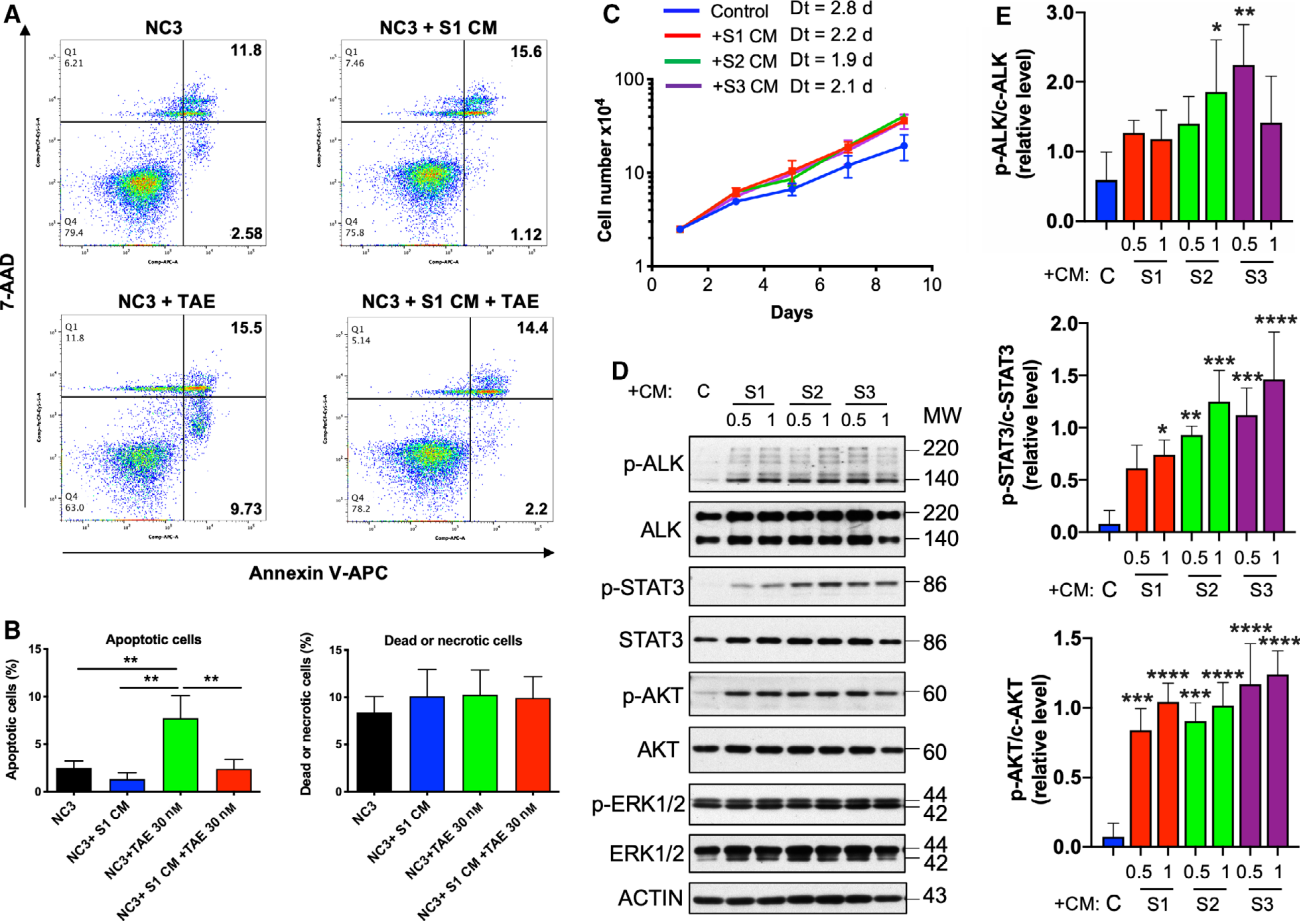
S‐type cell‐CM promotes NC3 cell proliferation and viability. (A) 3 × 10^5^ NC3 cells per well of 60‐mm plates were pre‐incubated with or without CM from S1 cells for 24 h and then were treated with 30 nm TAE684 for 48 h. Cells were stained with APC‐annexin V and 7‐AAD and analysed for fractions of AnV^+^ apoptotic cells (lower right quadrants) and AnV^+^ 7‐AAD^+^ dead or necrosis cells (upper right quadrants). (B) Results from three independent experiments were quantified using flowjo. Statistics, one‐way ANOVA. Error bars, SD. **P < 0.01. (C) Approximate 2 × 10^4^ NC3 cells were seeded per well of six‐well plates and were cultured with CM from S1, S2 and S3 cells or normal culture medium. Growth curves were obtained by counting cell numbers at indicated time points. Results from a single experiment performed in triplicates are shown on a semilog plot, and nonlinear regression analysis was performed to estimate the doubling time (Dt) in days (d). (D) 8 × 10^5^ NC3 cells per well of six‐well plates were exposed to S1, S2 and S3 CM diluted at the indicated ratio for 48 h. Cells were lysed for western blotting analysis with indicated antibodies. (E) The relative phosphorylation of the indicated signalling molecules was quantified from three independent experiments. Statistics, one‐way ANOVA. Error bars, SD. *P < 0.05. **P < 0.01. ***P < 0.001. ****P < 0.0001. [Colour figure can be viewed at wileyonlinelibrary.com]

To explore the protective effects of soluble factors secreted by the S‐type cells, the NC3 cells were cultured with or without CM from S1, S2 or S3 cells. A flow cytometry analysis showed that CMs from S1, S2 and S3 all significantly reduced the apoptotic cell populations in both the normal and TAE684‐treated conditions (Fig. 4A,B; Fig. S4). CM from S1 or S2 did not decrease the fraction of dead or necrotic cells. However, the CM from S3 had the ability to block NC3 cells from TAE684‐induced necrotic cell death (Fig. S4B,D). This result is consistent with previous experiments showing S3 cells exerted the strongest protective effect on NC3 cells under coculturing conditions.

Similar experiments were also conducted using the MTS cell viability assay. The N‐type cells were pre‐incubated with CM from S1, S2 or S3 for 24 h and then treated with an escalating concentration of TAE684 for 48 h. The CM from S1, S2 or S3 cells shifted the dose–response curve to the right and increased the IC_50_ by ~ 2.4‐fold, suggesting that this treatment had increased resistance to anti‐ALK treatment (Fig. S5A). The cell viabilities of the N‐type cells under different TAE684 concentrations are shown in Fig. S5B. The prosurvival effects of CM were only obvious in the 10–100 nm dose range. Furthermore, CM derived from S1, S2 or S3 cells increases in the proliferation rate and reduced the doubling time of the N‐type cells (Fig. 4C). In addition, CM from S1, S2 or S3 cells increased the levels of p‐ALK, p‐STAT3 and p‐AKT in the N‐type cells (Fig. 4D,E). The enhanced activation of ALK was not as strong as that for STAT3 and AKT, and as expected, S3 had a marginally greater capacity for stimulating prosurvival signalling. We further tested CM from S4 or S6 cell lines and both increased the survival and proliferation of NC3 cells treated with TAE684 (Fig. S1D,E). Both CM also stimulated the phosphorylation of STAT3 and AKT, albeit weaker when compared with S3 CM, especially for p‐STAT3. Therefore, in subsequent experiments, S3 cell line was selected for further analysis.

We next examined whether similar crosstalk existed in a different NB cell line, LA‐N‐1, which has *MYCN* amplification and *ALK* F1174L mutation [27]. LA1‐55n and LA1‐5s are N‐ and S‐type sublines of LA‐N‐1, respectively. LA1‐5s was relatively resistant to TAE684 treatment with only 40% suppression of cell survival at 300 nm TAE (Fig. S6A). However, it was sensitive to Stattic, a chemical inhibitor of STAT3. This inhibition was particularly prominent under low serum conditions at 0.1% FBS (Fig. S6A). The addition of CM from LA1‐5s significantly enhanced cell viability both at high and low serum conditions. LA1‐5s CM modestly stimulated STAT3, probably due to its constitutive phosphorylation. In contrast, it induced a robust activation of AKT (Fig. S6B). The p‐ERK1/2 level was undetectable in LA1‐55n. These results suggest that secreted factors from S‐type cells could promote survival in neuroblastic tumour cells harbouring *MYCN* and *ALK* oncogenes.

### 3.4 S‐type cells promote cell viability in N‐type cells in a STAT3‐dependent fashion

**Fig. 5:**
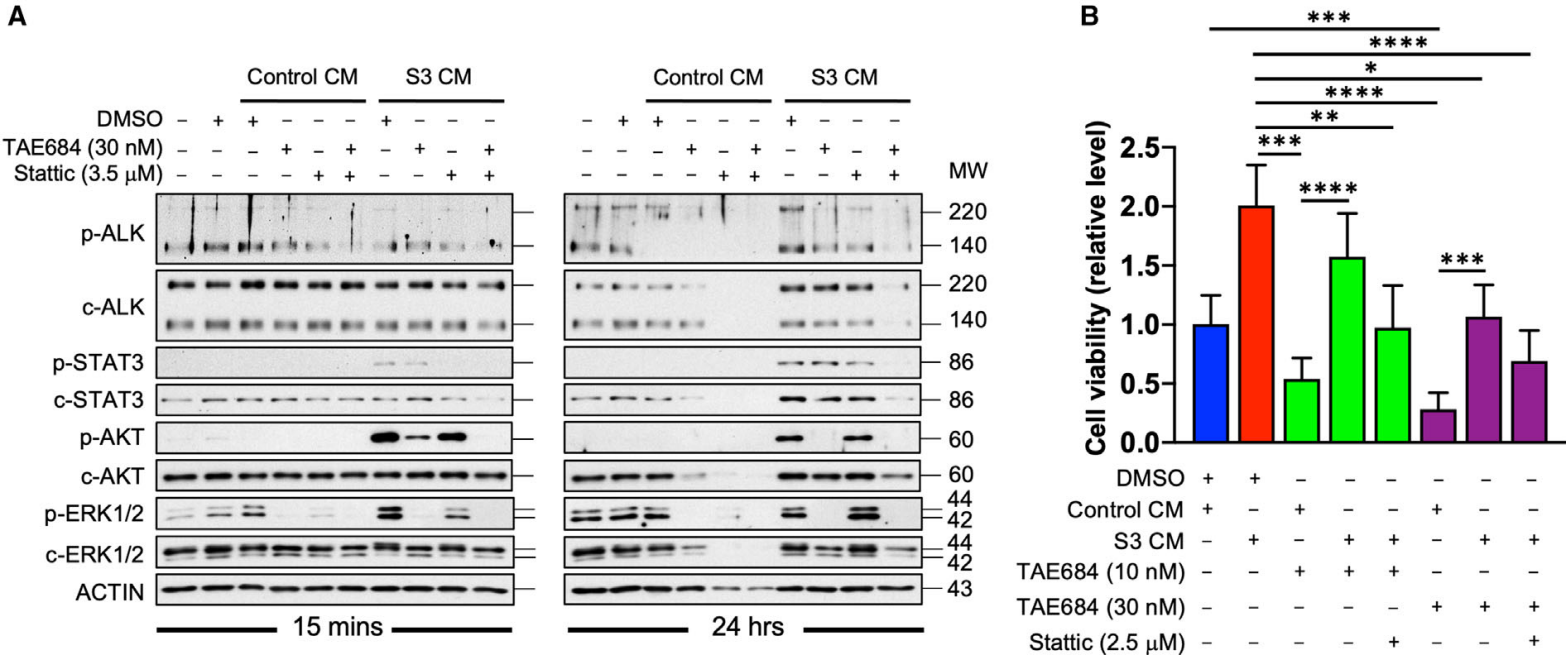
Dependence of STAT3 in mediating survival. (A) Approximately 2 × 10^5^ NC3 cells were seeded to each well of a 12‐well plate. Cells were treated with blank, control or S3 CMs in the absence or presence of combinations of TAE684 (30 nm) and Stattic (3.5 μm). Cell lysates were extracted after 15 min or 24 h of treatment, and western blotting analysis was conducted using the indicated antibodies. (B) Around 2 × 10^4^ NC3 cells were plated per well of a 96‐well plate in triplicates. Cells were treated with the indicated S3 CM and inhibitors for 3 days. Cell viability was measured by MTS assay, and results were quantified from five independent experiments. Statistics, one‐way ANOVA. Error bars, SD. *P < 0.05. **P < 0.01. ***P < 0.001. ****P < 0.0001. [Colour figure can be viewed at wileyonlinelibrary.com]

To address the role of ALK‐driven signalling pathways in enhancing N‐type cell survival promoted by CM from S‐type cells, the activation states of ALK, STAT3, AKT and ERK were monitored in the absence or presence of pathway inhibitors. As shown in Fig. 5A, following 15 min of stimulation by S3 CM, p‐STAT3, p‐AKT and p‐ERK levels were significantly increased and this activation was sustained for up to 24 h. The addition of TAE684, which suppressed ALK, drastically depleted p‐AKT and p‐ERK levels at the 15‐min timepoint but not those of p‐STAT3. These results suggest that it was unlikely that CM from S3 cells stimulates the STAT3 signalling pathway through the activation of ALK. Stattic, a small‐molecule inhibitor of STAT3, was able to eliminate the p‐STAT3 signal at the 15‐min timepoint but less so at the 24‐h timepoint, possibly due to reactivation of STAT3 (Fig. 5A). The complete abrogation of the CM‐induced p‐STAT3 at 24 h was achieved by combination treatment with TAE684 and Stattic, presumably due to drug‐induced cytotoxicity.

To test whether the prosurvival effects of CM could be abrogated by suppressing STAT3 signalling, MTS assays were carried out on NC3 cells exposed to a combination of TAE684 and Stattic in the presence or absence of CM. As expected, cell viability was clearly enhanced (by twofold) with CM from S3 (Fig. 5B). NC3 treated with 10 and 30 nm of TAE684 displayed a 50–75% loss of cell viability compared with control cells. The addition of S3 CM to TAE684‐treated NC3 enhanced cell viability by ~ 3‐fold. However, the addition of 2.5 μm Stattic reduced the prosurvival effects of S3 CM by ~ 40%. Overall, a combination of TAE684 and Stattic reduced cell viability by ~ 50–60% when compared with cells treated with S3 CM alone. These results indicate the potential therapeutic benefits of combined targeting of ALK and STAT3 in treating NB. Furthermore, the ability of S3 CM to confer survival on TAE684‐treated cells appears most likely to be mediated by an ALK‐independent paracrine activation of STAT3 in the N‐type cells. Therefore, isolating the soluble factors responsible for this activation would be warranted.

### 3.5 STAT3 activation mediated by S‐type cell CM is IL‐6‐independent

Various chemokines and cytokines have been reported to stimulate the STAT3 pathway. The mRNA expression levels of 18 cytokines were measured in both the N‐ and S‐type cells (Fig. S7A). Three of these cytokines, IL‐6, IL‐11 and IL‐15, were present in higher mRNA levels in the S‐type cells. The IL‐6 mRNA levels were 11 057‐, 31 838‐ and 76 296‐fold higher in S1, S2 and S3, respectively, when compared to NC3. The presence of IL‐6 in the S3 CM was confirmed by an ELISA. In the S3 CM, the IL‐6 protein concentration was approximately 0.29 ng·mL^−1^, while in CM derived from the NC3 cells, no IL‐6 protein was detected (Fig. S7B). The stimulation of signalling events was measured using increasing concentrations of recombinant human IL‐6 (rhIL‐6) over 15 min. As expected, rhIL‐6 activated STAT3 in a dose‐dependent manner (Fig. S7C). There was almost no change in AKT signalling subsequent to the various doses of rhIL‐6. A low dose of 0.6 ng·mL^−1^ rhIL‐6 resulted in the highest activation of ERK1/2 signalling. More importantly, the addition of rhIL‐6 at a concentration (0.29 ng·mL^−1^) found in the CM of S3 cells was ~ 25‐fold less effective in activating STAT3 when compared with S3 CM (Fig. S7C,D). Furthermore, IL‐6 at a concentration of 0.29 ng·mL^−1^ did not exert any detectable prosurvival activity in TAE684‐treated NC3 cultures (Fig. S7E). All these results suggested that additional secreted factors from S3 cells, other than IL‐6, were responsible for promoting cell survival in TAE684‐treated NC3 cells.

### 3.6 Transcriptomic profiling of the N‐ and S‐type cells by RNA‐Seq

**Fig. 6:**
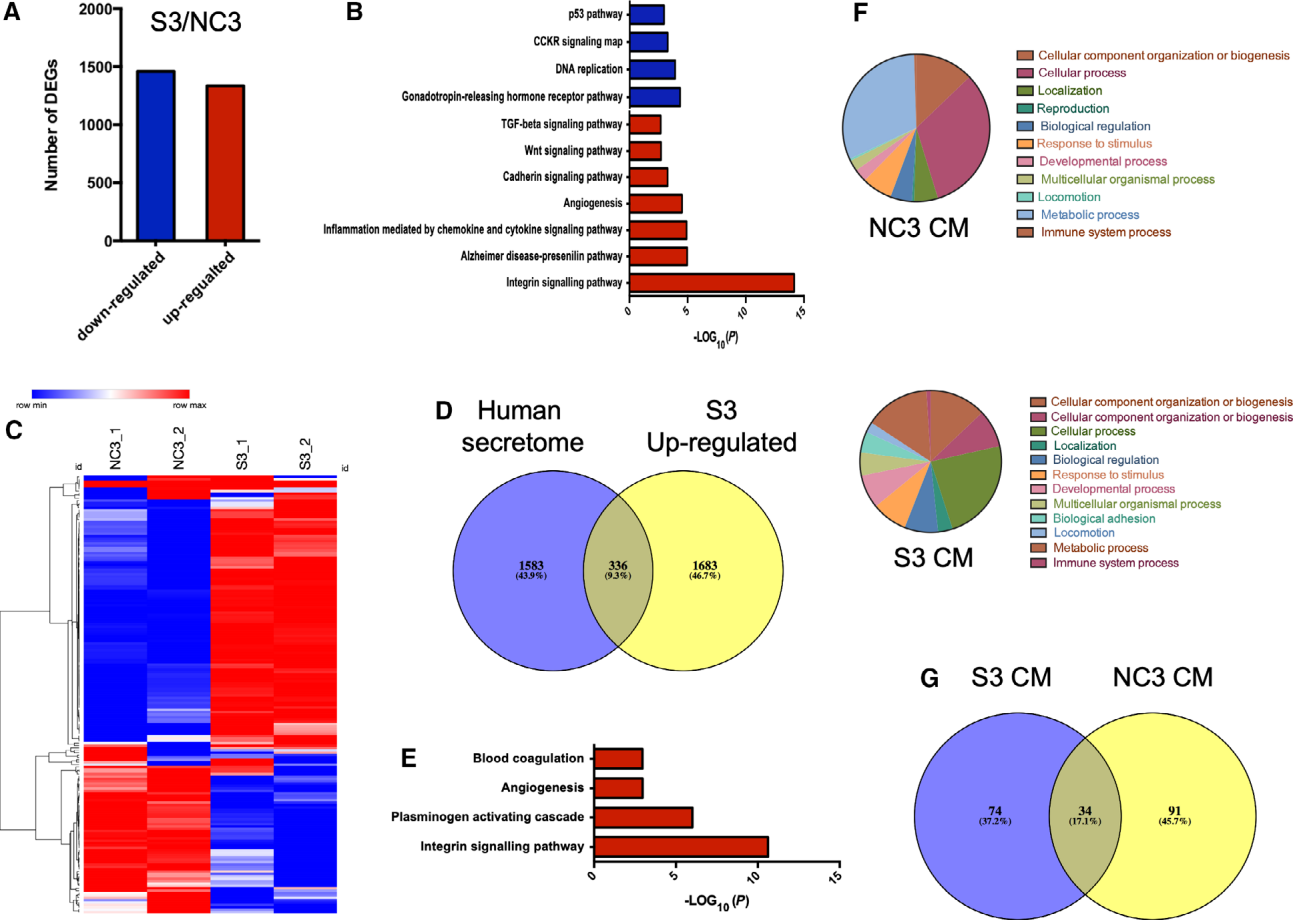
Transcriptomic and proteomic profiling of N‐ and S‐type cells. (A) RNA‐Seq analysis revealed DEGs between NC3 and S3 cells using the following thresholding criteria: fold change log_2_ > 2.0, *P*‐value < 0.05. (B) Pathway enrichment analysis was carried out based on database from Panther classification system (http://www.pantherdb.org/pathway/). (C) Integrins pathway‐related gene list was obtained from Panther pathway database. Heatmap was generated using an online tool from the Broad Institute (https://software.broadinstitute.org/morpheus/). Hierarchical clustering was based on one minus Pearson correlation. (D) DEGs upregulated in S3 were integrated with human secretome database from The Human Protein Atlas database identified 336 overlapping genes. Venn diagram was generated by an online tool Venny (http://bioinfogp.cnb.csic.es/tools/venny/index.html). (E) Pathway enrichment analysis of those 336 genes was based on Panther classification system. (F) GO enrichment analysis of identified proteins in NC3 and S3 CM based on mass spectroscopy studies. (G) Integration of the proteomes of S3 and NC3 CM identified 74 proteins unique for S3 cells. [Colour figure can be viewed at wileyonlinelibrary.com]

To identify differentially expressed genes (DEGs) in the S3, the transcriptomes of the NC3 and S3 cells were compared. A correlation analysis showed a high similarity between the two biological repeats (Fig. S8). Notably, the correlation between the NC3 and S3 cells was also high, probably because they were derived from the same progenitor cell. Analysis of these two transcriptomes revealed 1460 downregulated DEGs and 1334 upregulated DEGs in S3 cells, according to the thresholding criteria of fold change log_2_ > 2.0 and *P* < 0.05 (Fig. 6A). Pathway enrichment analysis based on the database from the PANTHER classification revealed that the upregulated DEGs in the S3 cells were highly enriched in the integrin signalling pathway (Fig. 6B). Other pathways that showed enrichment were TGF‐β, Wnt, cadherin and inflammation mediated by chemokine and cytokine signalling pathways. We further confirmed the gene expressions of the integrin signalling pathway‐correlated genes as present in four samples (Fig. 6C). About 70% of all integrin signalling pathway‐correlated genes were upregulated in the S‐type cells.

To identify secretory proteins in the S‐type cells, the upregulated DEGs in the S3 cells were integrated with the human secretome from the Human Protein Atlas database. We identified 336 common genes (Fig. 6D). These common genes also showed high enrichment in the integrin signalling pathway (Fig. 6E), suggesting a potential role of the integrin signalling pathway in the CM‐mediated signalling and prosurvival activities.

### 3.7 Identification of secreted proteins by mass spectroscopy

To directly identify the secreted factors from the S‐type cells, S3 cells were conditioned in serum‐free medium and proteins were subjected to mass spectroscopy. These analyses detected 108 and 125 proteins in the CM of S3 and NC3, respectively (Fig. 6F,G). The secretomes of the S3 and NC3 cells were distinct. Based on the Gene Ontology (GO) database, the proteins from both the NC3 CM and S3 CM were mainly involved in cellular and metabolic processes, but S3 CM harboured more proteins related to biological adhesion (Fig. 6F). A total of 74 proteins specifically secreted in the S3 cells were identified (Fig. 6G).

**Fig. 7:**
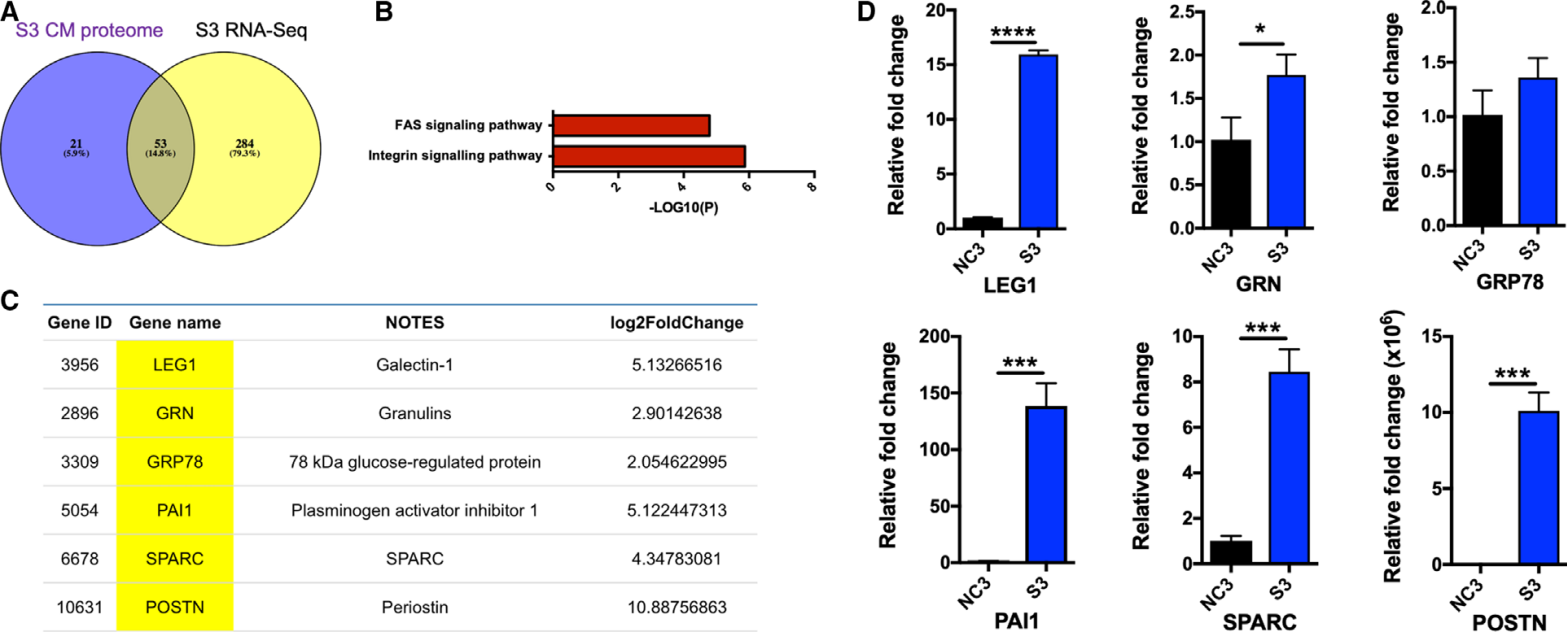
Identification of prosurvival factors from S3 cells. (A) Integration of S3‐specific secreted factors identified by transcriptomic and proteomic approaches identified 53 common genes. (B) Pathway enrichment analysis of the 53 genes. (C) Six selected genes associated with STAT3 pathway with their Gene ID, symbol, description and gene expression fold change from RNA‐Seq data being shown. (D) mRNA expression levels of six candidate genes were determined by real‐time qRT‐PCR using the 2^−ΔΔCT^ method. Data were from three technical replicates. Results were analysed with Student's *t*‐test. Error bars, SD. *P < 0.05. ***P < 0.001. ****P < 0.0001. [Colour figure can be viewed at wileyonlinelibrary.com]

Several proteins in the list had a well‐established role in tumour development. For example, the secreted frizzled‐related protein 1 (sFRP1) is a negative regulator of the Wnt signalling pathway through the direct interaction with Wnts. This protein has been reported to mediate cell proliferation, cell differentiation and angiogenesis in tumours [28]. Unexpectedly, IL‐6 was not identified in the S3 CM. We further integrated the RNA‐Seq dataset with the proteomic dataset and found 53 common genes (Fig. 7A and Table S1). These genes showed an enrichment in Fas and integrin signalling pathways (Fig. 7B).

To further narrow down the candidate list, we selected six genes based on their reported ability to activate the STAT3 signalling pathway (Fig. 7C). These included *POSTN* [29], *LEG1* [30], plasminogen activator inhibitor 1 (*PAI1*) [31], *SPARC* [32, 33], granulins (*GRN*) [34] and glucose‐regulated protein 78 (*GRP78*) [35, 36]. We validated the mRNA expression levels of the selected genes. *LEG1*, *PAI1*, *SPARC* and *POSTN* showed significantly higher expression levels in the S3 cells. The mRNA expressions of *GRP78* and *GRN* were slightly increased in the S‐type cells (Fig. 7D). The normalized fold changes in the *POSTN*, *LEG1*, *PAI1* and *SPARC* gene expression from the RNA‐Seq data were higher than those in *GRN* and *GRP78* (Fig. 7C, right panel). We therefore selected these four factors for functional analysis.

**Fig. 8:**
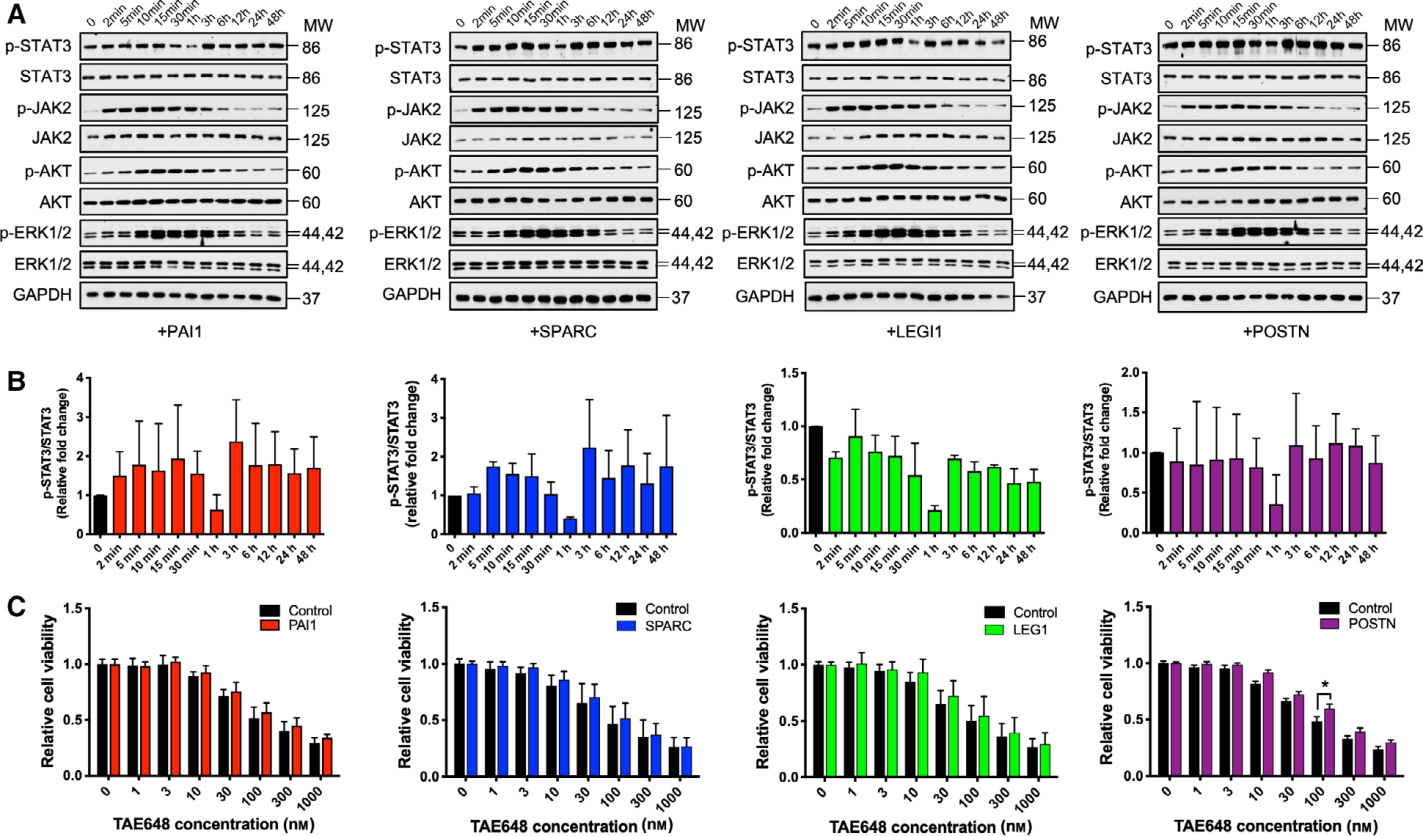
Functional characterization of candidate proteins secreted from S3 cells. (A) 5 × 10^5^ NC3 cells per well of six‐well plates were incubated with human recombinant PAI1 protein (200 ng·mL^−1^), SPARC protein (500 ng·mL^−1^), LEG1 protein (200 ng·mL^−1^) or POSTN protein (500 ng·mL^−1^) for the indicated period. Cell lysates were collected and subjected to western blotting analysis using the indicated antibodies. (B) The relative phosphorylation of STAT3 signalling molecules was analysed from two independent experiments using one‐way ANOVA. Error bars, SD. (C) Around 2 × 10^5^ N‐type cells were plated per well of 96‐well plates in triplicates and were pre‐incubated with or without PAI1 (200 ng·mL^−1^), SPARC (500 ng·mL^−1^), LEG1 (200 ng·mL^−1^) or POSTN (500 ng·mL^−1^) for 24 h, then were treated with an increasing concentration of TAE684 for 48 h. Cell viability was measured by the MTS assay. Results from three independent experiments were analysed using two‐way ANOVA. Error bars, SD. *P < 0.05. [Colour figure can be viewed at wileyonlinelibrary.com]

LEG1, PAI1, SPARC and POSTN recombinant proteins were tested for their ability to stimulate signalling pathways in the N‐type cells. Human recombinant proteins for these factors were added to the NC3 cells over time. The kinetics of STAT3 activation induced by these secreted factors was akin to that of IL‐6, being biphasic in nature (Fig. 8A). However, only PAI1 and SPARC showed stimulation of STAT3 signalling (Fig. 8B), suggesting that there is a potential role played by PAI1 and SPARC in promoting the survival and proliferation of the N‐type cells. Notably, these four factors were all able to activate other signalling pathways, including the JAK2, AKT and ERK1/2 signalling pathways. Finally, all four factors were partially able to enhance cell viability towards TAE684 treatment with POSTN, displayed statistically significant results in cells treated with 100 ng·mL^−1^ of TAE684 (Fig. 8C).

## 4 Discussion

We identified a subpopulation of substrate‐adherent cells that can protect neuroblastic tumour cells from an ALK kinase inhibitor, TAE684 [37]. The cell line SK‐N‐SH was derived from a bone marrow metastasis of a 4‐year‐old girl, but whether the ALK F1174L mutation in the SK‐N‐SH was germline or *de novo* is unknown. We assumed that the N‐ and S‐type cells isolated in this study were derived from the same clonal origin as they both harboured the same ALK mutation. The passage 35 SK‐N‐SH cell line we used had ~ 74% S‐type cells. Instead, most published studies using SK‐N‐SH have a higher proportion of neuroblastic N‐type cells [38, 39]. This may be due to the higher proliferative rate of N‐type cells over S‐type cells during the propagation of this cell line in different laboratories. It is also possible that a higher fraction of S‐type cells may establish a favourable metastatic niche for neuroblastic tumour cells in the bone marrow microenvironment.

Our data demonstrated the protective effects of S‐type cells by coculture or CM approaches. Both treatments reduced early apoptotic populations, but CMs were less able to rescue necrotic cell death. The tumour–stroma interaction occurred through direct cell–cell interactions and paracrine mechanisms [40]. Using CM, we eliminated the effects of cell–cell interaction. Thus, the inability of CM to protect N‐type cells from necrotic cell death suggests that the cell–cell interactions are more critical in preventing early apoptosis.

We observed that ALK in N‐type cells could be further upregulated by CM from S‐type cells. This raises the possibility that an ALK ligand is secreted by S‐type cells. We also observed that CM from S‐type cells could stimulate STAT3, AKT and ERK1/2. Interestingly, TAE684 was effective in blocking AKT and ERK1/2 activation, but not STAT3. This implied that ALK actions are mediated mainly through AKT and ERK1/2 in N‐type cells of SK‐N‐SH. Therefore, our data imply that factors secreted by S‐type cells can stimulate STAT3 in an ALK‐independent manner. We also found that inhibiting STAT3 partially reduced the protective effect of CM derived from S‐type cells.

The role of the IL‐6/STAT3 signalling axis in proliferation, cell survival, cell invasiveness and metastasis of NB is well established. Based on our data, the level of IL‐6 detected in the CM of S3 would not be sufficient to account for the full activation of STAT3. However, we cannot exclude the possibility that the IL‐6 secreted by S‐type cells might be far more active because of post‐translational modifications or that low levels of IL‐6 can act synergistically with other soluble factors to activate STAT3. Our attempt to identify STAT3‐activating soluble factors led to the isolation of multiple candidates. One limitation of our purification approach was that the molecular weight cut‐off of the concentration column was 3 kDa, which meant that factors smaller than that would have been excluded from our samples. Also, we have only included factors that had been previously linked to the STAT3 signalling pathway. Another limitation is that transcriptome data only reflect changes in mRNA levels and proteins synthesized may not be even secreted. Conversely, there may be similar expression levels of mRNA and the difference is that S3 cells secrete them.

Notably, a high fraction of the secreted factors from S‐type cells are part of the integrin signalling pathway. Integrins serve as transmembrane surface receptors linking the extracellular matrix to intracellular cytoskeletal linker proteins. The integrin signalling pathway also mediates growth factor‐induced signalling and regulates cellular processes such as cell proliferation and survival [41]. Additional mining of our datasets would be necessary to identify other candidates.

Based on our approach, four genes were selected: *POSTN*, *LEG1*, *PAI‐1* and *SPARC*. POSTN is a secreted extracellular matrix protein that is involved in tissue regeneration and development. It binds to integrins to regulate epithelial cell adhesion and migration, which suggests that it has an important role in tumour metastasis [42]. In breast cancers, LEG1 activates the downstream focal adhesion kinase/c‐Src pathway, which further stimulates ERK and STAT3 signalling leading to enhanced expression of survivin, and inhibits apoptosis and conferred drug resistance [30]. The PAI‐1 has been shown to promote cell proliferation and cell migration. Recently, the PAI‐1/PIAS3/Stat3/miR‐34a axis has been reported to regulate tumour metastasis in NSCLC [31]. PAI‐1 interacts with PIAS3 to activate the STAT3 signalling pathway, regulating STAT3‐dependent gene expression and suppressing miR‐34a.

The SPARC is also a matrix‐associated protein. In medulloblastoma, SPARC overexpression decreased STAT3 phosphorylation, leading to cell cycle arrest and neuronal differentiation [32, 33]. SPARC has also been reported to activate STAT3 and promote cell proliferation and adhesion [43]. Our results demonstrated that PAI1 and SPARC induced activation of STAT3, and all four proteins activated AKT and ERK1/2 pathways. Moreover, all four proteins partially enhanced cell viability. We speculate that a certain combination of these proteins, in addition to IL‐6, may confer greater cell survival to TAE684‐treated cells.

## 5 Conclusion

Based on a detailed investigation of a well‐established NB cell line, SK‐N‐SH, we uncovered diversity in cellular and molecular subtypes covering a range of developmental lineages and can be broadly categorized into ALK‐positive neuroblastic tumour cells and tumour‐derived ALK‐negative substrate‐adherent cells. Secretory factors from S‐type cells promote survival and proliferation of neuroblastic tumour cells in a STAT3‐dependent manner. Integrating transcriptomic and proteomic data from S‐type cells identified a combination of PAI1, SPARC, POSTN and LEG1 soluble factors to exert survival promoting effects. Taken together, our findings suggest that the targeting of tumour‐derived S‐type cells could enhance the efficacy of targeted therapeutic drugs in ALK‐positive NB.

## Conflict of interest

The authors declare no conflict of interest.

## Author contributions

JL and AMC conceived the idea and designed the experiments. JL, YW, LL, PM‐YO, CWW, TL and WLHH conducted the cell based and omics studies. JL and AMC wrote the manuscript.

## Supporting information


**Fig. S1**. Characterization of SK‐N‐SH derived S‐type cells.
**Fig. S2**. S‐type cells protect NC3 cells from apoptosis by co‐cultivation.
**Fig. S3**. LA1‐5s cells promote the viability of NC3 cells from apoptosis by co‐cultivation.
**Fig. S4**. S‐type cell CM promotes NC3 cell viability.
**Fig. S5**. S‐type cell CM promotes cell viability in TAE684‐treated NC3 cells.
**Fig. S6**. LA1‐5s CM promotes cell viability in LA1‐55n cells.
**Fig. S7**. Activation of STAT3 signaling by IL‐6 in NC3 cells.
**Fig. S8**. RNA‐Sequencing analysis of S3 and NC3 cells.
**Table S1**. Candidate gene list.

## Data Availability

The RNA‐Seq datasets generated in this study have been deposited to the Gene Expression Omnibus (GEO) at the NCBI with the accession no: GSE139235.
