# Supplementary material for: Tumour‐derived substrate‐adherent cells promote neuroblastoma survival through secreted trophic factors
Source: Mol Oncol. 2021 May 6;15(8):2011–25. doi: 10.1002/1878-0261.12969 (PMC8334291; doi:10.1002/1878-0261.12969)
Supplement: Supplementary file 1 — Fig. S1. Characterization of SK‐N‐SH derived S‐type cells. Fig. S2. S‐type cells protect NC3 cells from apoptosis by co‐cultivation. Fig. S3. LA1‐5s cells promote the viability of NC3 cells from apoptosis by co‐cultivation. Fig. S4. S‐type cell CM promotes NC3 cell viability. Fig. S5. S‐type cell CM promotes cell viability in TAE684‐treated NC3 cells. Fig. S6. LA1‐5s CM promotes cell viability in LA1‐55n cells. Fig. S7. Activation of STAT3 signaling by IL‐6 in NC3 cells. Fig. S8. RNA‐Sequencing analysis of S3 and NC3 cells. Table S1. Candidate gene list. [file MOL2-15-2011-s001.docx]

Supporting Information

**Tumour-derived substrate-Adherent cells promote neuroblastoma survival through secreted trophic factors**

Jing Li, Yubing Wang, Lisha Li, Penelope M.-Y. Or, Chi Wai Wong, Tian Liu, Wayne L.H. Ho and Andrew M. Chan

**Table S1.** Candidate gene list

**Figure S1.** Characterization of SK-N-SH derived S-type cells

**Figure S2.** S-type cells protect NC3 cells from apoptosis by co-cultivation

**Figure S3.** LA1-5s cells promote the viability of NC3 cells from apoptosis by co-cultivation

**Figure S4.** S-type cell conditioned medium (CM) promotes NC3 cell viability.

**Figure S5.** S-type cell conditioned medium (CM) promotes cell viability in TAE684-treated NC3 cells

**Figure S6.** LA1-5s conditioned medium (CM) promotes cell viability in LA1-55n cells

**Figure S7.** Activation of STAT3 signaling by IL-6 in NC3 cells.

**Figure S8.** RNA-Sequencing analysis of S3 and NC3 cells.

**Table S1** Candidate gene list

| **Gene ID** | **Symbol** | **Description** | **Log2 fold change** |
| --- | --- | --- | --- |
| 83872 | *HMCN1* | Hemicentin-1 | 12.1603369 |
| 2202 | *FBLN3* | EGF-containing fibulin-like extracellular matrix protein | 12.1447187 |
| 7045 | *BGH3* | Transforming growth factor-beta-induced protein ig-h3 | 11.4589126 |
| 10631 | *POSTN* | Periostin | 10.8875686 |
| 1277 | *CO1A1* | Collagen alpha-1(I) chain | 10.6077294 |
| 633 | *PGS1* | Biglycan | 8.96030696 |
| 4052 | *LTBP1* | Latent-transforming growth factor beta-binding protein 1 | 8.61123283 |
| 10085 | *EDIL3* | EGF-like repeat and discoidin I-like domain protein 3 | 8.28260912 |
| 4060 | *LUM* | Lumican | 8.12980386 |
| 1281 | *CO3A1* | Collagen alpha-1(III) chain | 7.8055798 |
| 7057 | *TSP1* | Thrombospondin-1 | 7.55839281 |
| 3624 | *INHBA* | Inhibin beta A chain | 7.53692323 |
| 7058 | *TSP2* | Thrombospondin-2 | 6.98980906 |
| 5654 | *HTRA1* | Serine protease HTRA1 | 6.30859634 |
| 1893 | *ECM1* | Extracellular matrix protein 1 | 5.61170492 |
| 51200 | *CBPA4* | Carboxypeptidase A4 | 5.4567164 |
| 9358 | *ITGBL* | Integrin beta-like protein 1 | 5.38866093 |
| 5054 | *PAI1* | Plasminogen activator inhibitor 1 | 5.12244731 |
| 6422 | *SFRP1* | secreted frizzled related protein 1 | 5.04682831 |
| 2335 | *FINC* | Fibronectin | 4.84248692 |
| 2934 | *GELS* | Gelsolin | 4.8260828 |
| 11167 | *FSTL1* | Follistatin-related protein 1 | 4.78751623 |
| 1634 | *PGS2* | Decorin | 4.73644738 |
| 80781 | *COIA1* | Collagen alpha-1(XVIII) chain | 4.57275092 |
| 5806 | *PTX3* | Pentraxin-related protein PTX3 | 4.43971692 |
| 6678 | *SPRC* | SPARC | 4.34783081 |
| 1462 | *CSPG2* | Versican core protein | 4.20924025 |
| 30008 | *FBLN4* | EGF-containing fibulin-like extracellular matrix protein | 3.95266315 |
| 3339 | *PGBM* | Basement membrane-specific proteoglycan core protein | 3.85861902 |
| 7076 | *TIMP1* | Metalloproteinase inhibitor 1 | 3.67592903 |
| 4239 | *MFAP4* | Microfibril-associated glycoprotein 4 | 3.24619636 |
| 4811 | *NID1* | Nidogen-1 | 3.21179043 |
| 3487 | *IBP4* | Insulin-like growth factor-binding protein 4 | 3.07688451 |
| 871 | *SERPH* | Serpin H1 | 3.05456113 |
| 1363 | *CBPE* | Carboxypeptidase E | 3.04578295 |
| 5351 | *PLOD1* | Procollagen-lysine,2-oxoglutarate 5-dioxygenase 1 | 2.90729165 |
| 2896 | *GRN* | Granulins | 2.90142638 |
| 1200 | *TPP1* | Tripeptidyl-peptidase 1 | 2.81767832 |
| 1291 | *CO6A1* | Collagen alpha-1(VI) chain | 2.80853085 |
| 1471 | *CYTC* | Cystatin-C | 2.6120297 |
| 3073 | *HEXA* | Beta-hexosaminidase subunit alpha | 2.5784297 |
| 1509 | *CATD* | Cathepsin D | 2.29997211 |
| 3309 | *GRP78* | 78 kDa glucose-regulated protein | 2.05462299 |
| 9601 | *PDIA4* | Protein disulfide-isomerase A4 | 2.04354985 |

**Fig. S1.** Characterization of SK-N-SH derived S-type cells. (A) Brightfield photomicrographs of subconfluent passage 35 (P35) SK-N-SH. N-type (red arrows) and S-type (green marks) cells are indicated. *Bar*, 100 μm. (B) The relative N- and S-type cells are quantified from 13 randomly selected fields. *Bars*, means. (C) ALK genomic regions from S4 and S6 cells were sequenced to reveal the C to A substitution at codon 1174 (red arrows). (D) Around 2x10^4^ NC3 cells were plated per well of 96-well plates in triplicates and were incubated with or without CM from S4 or S6 cells, and then treated with an increasing concentration of TAE684 for 48 hrs. Cell viability was measured by the MTS assay. *Error bars*, SD. Results were from 3 independent experiments with estimated IC50 values shown. (E) Around 1x10^5^ NC3 cells were plated per well of 12-well plates in triplicates and were incubated with or without CM from S4 or S6. Cells were treated with or without TAE684 (50 nM) for two days. Cells were counted in triplicates from a single experiment. (F) Around 1.5x10^5^ NC3 cells per well of 12-well plates were incubated with control blank (*C*) CM or CM from S3, S4, or S6 cells for the indicated durations. Western blotting analysis was carried out using the antibodies indicated.

**Fig. S2.** S-type cells protect NC3 from apoptosis by co-cultivation. 3x10^5^ NC3 cells per well of 60-mm plates were co-mixed with or without 1x10^5^ CFSE-labeled S2 (A) or S3 (C) cells and were treated with 30 nM TAE684 for 48 hrs. Cells were stained with APC-annexin V and 7-AAD. CFSE-negative populations (NC3 cells) were analyzed for fractions of AnV+ apoptotic cells (lower right quadrants) and AnV+ 7-AAD+ dead or necrosis cells (upper right quadrants). (B,D*)* Results from 3 independent experiments were quantified using FlowJo. Statistics, Two-way ANOVA. *Error bars*, SD. *, *p* < 0.05; **, *p*< 0.01; ***, *p*<0.001; ****, *p*<0.0001.

**
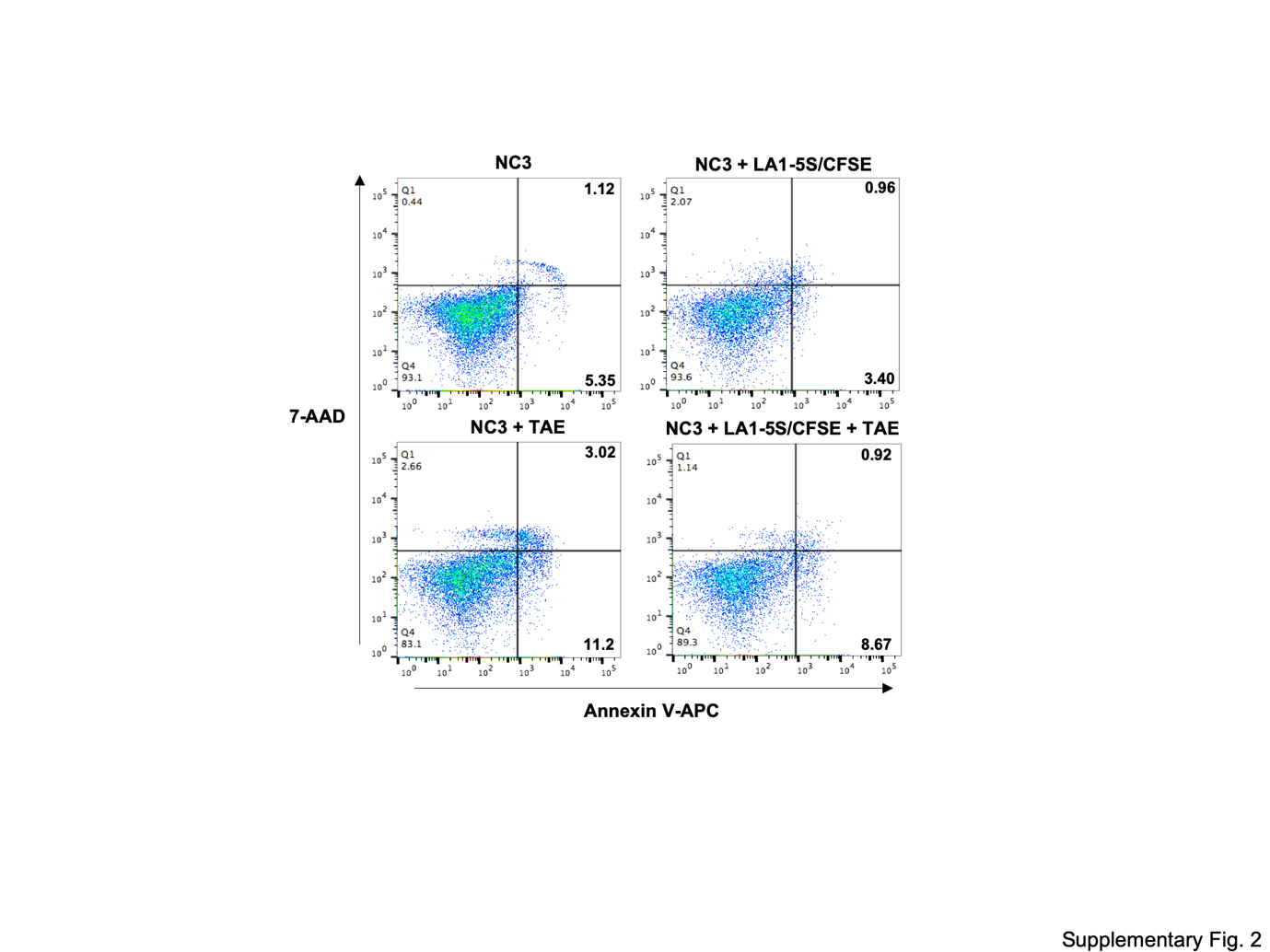
**

**Fig. S3.** LA1-5S cells protect NC3 cells from TAE684-induced apoptosis by co-cultivation. 3x10^5^ NC3 cells per well of 60-mm plates were co-mixed with or without 1x10^5^ CFSE-labeled LA1-5S cells and were treated with 30 nM TAE684 for 48 hrs. Cells were stained with APC-annexin V and 7-AAD. CFSE-negative populations (N-type cells) were analyzed for fractions of AnV+ apoptotic cells (lower right quadrants) and AnV+ 7-AAD+ dead or necrosis cells (upper right quadrants).

**Fig. S4.** S-type cell conditioned medium (CM) promotes N-type cell viability. 3x10^5^ NC3 cells per well of 60-mm plates were pre-incubated with or without CM from S2 (A) and S3 (B) cells for 24 hrs, and then were treated with 30 nM TAE684 for 48 hrs. Cells were stained with APC-annexin V and 7-AAD and analyzed for fractions of AnV^+^ apoptotic cells (lower right quadrants) and AnV^+^ 7-AAD^+^ dead or necrosis cells (upper right quadrants). (C, D) Results from 3 independent experiments were quantified using FlowJo. One-way ANOVA. *Error bars*, SD. *, *p* <0.05; **, *p*<0.01; ****, *p*<0.0001.

**Fig. S5.** S-type cell conditioned medium (CM) promotes cell viability in NC3 cells treated with TAE684. (A) Around 2x10^4^ NC3 cells were plated per well of a 96-well plate in triplicates and were pre-incubated with or without CM from S1, S2, and S3 cells for 24 hrs, then were treated with an increasing concentration of TAE684 for 48 hrs. Cell viability was measured by the MTS assay. *Error bars*, SD. (B) Statistic results of cell viability at each TAE684 dose were shown. Results were from 3 independent experiments. Two-way ANOVA. Error bars, SD. *, *p* < 0.05; **, *p* < 0.01; ****, *p* <0.0001.

**Fig. S6.** LA1-5s conditioned medium (CM) promotes cell viability in LA1-55n cells. (A) LA1-5s cells were conditioned with 10% or 0.1% FBS for 48 hrs. Around 1x10^4^ LA1-55n cells were plated per well of 96-well plates in triplicates were pre-incubated with either control or LA1-5s CM for 1 hr. Cells were then treated with an increasing concentration of TAE684 for 48 hrs under 10% or 0.1% FBS. Cell viability was measured by the MTS assay. Results were from 5 independent experiments. *Error bars*, SD. Mann-Whitney test. *, *p* < 0.05; **. (B) Around 1.5x10^5^ LA1-55n cells per well of 12-well plates were treated with LA1-5s CM for the indicated time in 10% or 0.1% FBS. Cells were solubilized and Western blotting analysis was carried out using the indicated antibodies.

**Fig. S7.** Activation of STAT3 signaling by IL-6 in NC3 cells. (A) mRNA levels of 18 cytokines and chemokines in NC3, S1, S2 and S3 cells were quantified by real time quantitative PCR using the 2^-ΔΔCT^ method. (B) NC3 and S3 cells were cultured to near confluence in 10% FBS/DMEM and CM was collected. IL-6 concentration was measured using an IL-6 ELISA kit (BD Biosciences). (C) 5x10^5^ NC3 cells per well of a 6-well plate were incubated with recombinant human IL-6 (rhIL-6) protein at 10 ng/ml for the indicated period. Cell extracts were prepared, and Western blotting analysis was conducted using the indicated antibodies for various signaling molecules. (D) The relative phosphorylation of the indicated signaling molecules was analyzed from 3 independent experiments using One-way ANOVA. Error bars, SD. *p< 0.05, **p< 0.01. (E) Cell viability assay was carried out as described in the legend of Fig. 5B. Results quantified from 6 independent experiments. *Error bars*, SD. **** p<0.0001; ns, not significant.


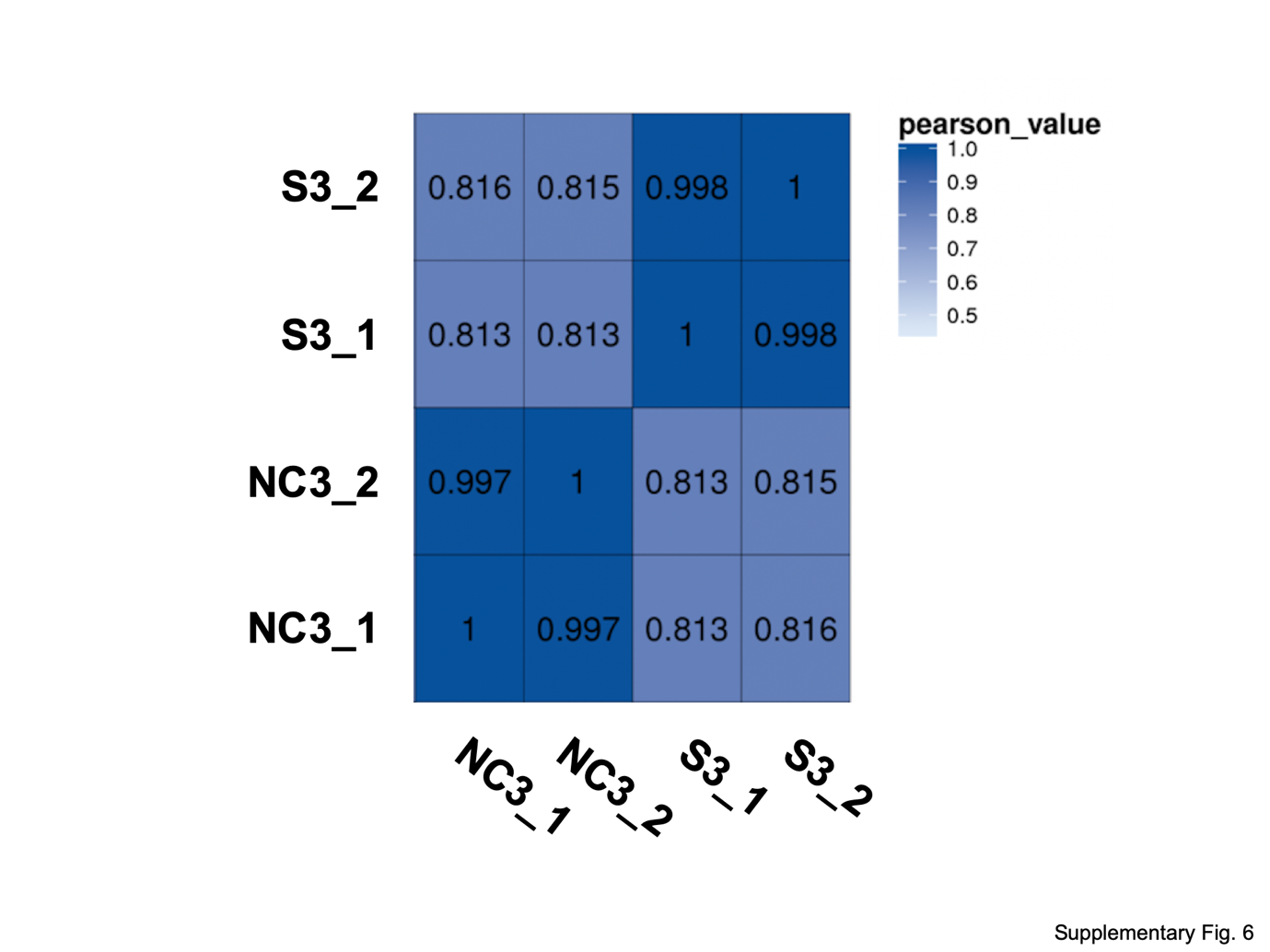


**Fig. S8.** RNA-Sequencing analysis of NC3 and S3 cells. Pearson correlation coefficients for all gene expression levels between each two samples were calculated and showed as heatmap. The X and Y axis represented each sample. The color represented the correlation coefficient (the darker the color, the higher the correlation).
